# Dendrimer-Conjugated Glutamine Antagonist, D-TTM020, Ameliorates Brain Immune Dysregulation and Improves Neurobehavioral Deficits in the *Mecp2-*Deficient Mouse Model

**DOI:** 10.3390/cells15030272

**Published:** 2026-02-01

**Authors:** Preeti Vyas, Elizabeth Smith Khoury, Nirnath Sah, Anjali Sharma, Javier Allende Labastida, Elizabeth L. Wilkinson, Kathleen Lac, Nerketa N. L. Damiba, Amanda Fowler, Jinhuan Liu, Ashley Bedner, Pavel Majer, Tomás Tichý, Ajit G. Thomas, Rana Rais, Barbara S. Slusher, Rangaramanujam M. Kannan, Sujatha Kannan

**Affiliations:** 1 Department of Anesthesiology and Critical Care Medicine, Johns Hopkins University School of Medicine, 1800 Orleans St, Baltimore, MD 21287, USA; pvyas2@jhmi.edu (P.V.); essmith1215@gmail.com (E.S.K.); nirnathsah@gmail.com (N.S.); jallend3@jhmi.edu (J.A.L.); nellylaurendamb@gmail.com (N.N.L.D.); amanda.fowler638@gmail.com (A.F.); jliu5@jhmi.edu (J.L.); asammmm88@gmail.com (A.B.); 2 Center for Nanomedicine, Wilmer Eye Institute, Department of Ophthalmology, Johns Hopkins University School of Medicine, Baltimore, MD 21205, USA; anjali.sharma@wsu.edu (A.S.); e.leigh.wilkinson@gmail.com (E.L.W.); krangar1@jhmi.edu (R.M.K.); 3 Department of Chemical and Biomolecular Engineering, Johns Hopkins University, Baltimore, MD 21218, USA; 4 Institute of Organic Chemistry and Biochemistry, Czech Academy of Sciences, Flemingovo n. 2, 160 00 Prague, Czech Republic; pavelm18@yahoo.com (P.M.); tomas.tichy@uochb.cas.cz (T.T.); 5 Johns Hopkins Drug Discovery, Johns Hopkins University School of Medicine, Baltimore, MD 21205, USA; ajit.thomas@jhmi.edu (A.G.T.); rrais2@jhmi.edu (R.R.); bslusher@jhmi.edu (B.S.S.); 6 Department of Neurology, Johns Hopkins University, Baltimore, MD 21218, USA; 7 Department of Biomedical Engineering, Johns Hopkins University, Baltimore, MD 21218, USA

**Keywords:** Rett Syndrome, glutaminergic pathway, D-TTM020, dendrimer, nanotherapy, nanomedicine, glutaminase, microglia, targeted therapy, neurobehavioral studies

## Abstract

Rett Syndrome (RTT) is a neurodevelopmental disorder characterized by mutations in the MeCP2 gene, predominantly affecting females. Recent work with MeCP2-deficient mouse models showed a significant role in glutamatergic transmission, specifically microglia-produced glutamate and glutaminase upregulation, in RTT pathology. The glutamine antagonist 6-diazo-5-oxo-L-norleucine (DON) is a potent glutaminase inhibitor; however, its use is limited due to systemic toxicities arising from its non-specific inhibition of glutamine-utilizing reactions. In this work, we determined whether dendrimer conjugation of a DON analog, TTM020 (or D-TTM020), results in targeted microglial glutaminase inhibition and behavioral changes in *Mecp2* KO and heterozygous mice upon systemic administration. D-TTM020 at 1 mg/kg (drug basis) selectively and significantly inhibits glutaminase enzyme activity in the microglia of *Mecp2* KO mice. Biweekly systemic treatment with 1 mg/kg of D-TTM020 improved the neurobehavioral phenotype in symptomatic *Mecp2* KO and het mice. D-TTM020 also restored long-term retrieval of conditioned fear memory and improved cue responses during fear extinction after 8 weeks of treatment in symptomatic *Mecp2* het mice. Our data indicate that selectively targeting glutamine metabolism in dysregulated glia using dendrimers represents a promising strategy that may offer a therapeutic approach for addressing glutamate dysregulation in RTT.

## 1. Introduction

Rett Syndrome (RTT), a rare developmental disorder, is characterized by mutations in the *Mecp2* gene and affects approximately 1 in 10,000 females [1,2,3]. The clinical manifestations of RTT encompass a range of symptoms, including respiratory difficulties, seizures, repetitive hand movements, motor impairments, intellectual disability, and additional complications. Over the past several years, there have been substantial advancements in the care of these children [4]. While trofinetide has recently received FDA approval for RTT [5,6,7,8], there is still an urgent need for more targeted therapeutic strategies. Rett patients and mouse models of RTT demonstrate glial dysfunction, immune-system dysregulation with disrupted cytokine signaling, redox imbalance, increased oxidative stress, and dysregulation of glutamatergic pathways with high glutamine and extracellular brain glutamate levels [9,10,11,12]. Recent studies suggest the involvement of glutathione, overexpression of glutaminase, and altered glutamate clearance in Rett microglia [9,11,13,14,15]. Enhancing microglial function and suppressing overactive glutaminergic pathways by targeting glutaminase with a potent glutamine antagonist can reduce excitotoxicity and attenuate injury. However, glutaminase and other glutamine-regulated enzymes are ubiquitous and involved in normal brain and cellular function [16]. Therefore, specifically inhibiting glutaminase in dysregulated glia may be a more effective mechanism to decrease glutamate toxicity to neurons without impairing normal glutaminase function.

6-Diazo-5-oxo-L-norleucine (DON), a potent first-generation glutamine antagonist and glutaminase inhibitor, has long demonstrated potential as an anticancer agent by effectively blocking tumor metabolism and halting cancer-cell proliferation [17,18,19,20]. It disrupts the tricarboxylic-acid cycle by blocking the conversion of glutamine to α-ketoglutarate, which starves cells of energy [19]. However, its clinical application was historically restricted due to dose-limiting adverse effects, including severe gastrointestinal toxicities (nausea, vomiting, diarrhea, and mucositis) and systemic adverse effects resulting from non-specific glutaminase inhibition and its distribution into healthy tissues [17,18,21]. Interventions that can specifically target dysregulated glutamatergic signaling in the glia can overcome these challenges and hold clinical promises. It can drastically limit exposure to healthy, non-target organs, thereby mitigating the severe GI and systemic toxicities of DON and ultimately enabling its safe delivery.

We have previously shown that hydroxyl-terminated poly(amidoamine) (PAMAM-OH) dendrimers, which are scalable, can overcome adverse effects and limitations in brain penetration [22,23,24,25]. It is biocompatible, non-toxic, ~4 nm, and 14 kD, and targets activated microglia upon systemic administration [22,23,26]. Beyond our own work, it has been established that these neutral nanoparticles avoid the aggressive blood–component interactions seen with cationic versions [27,28,29] and effectively bypass the blood–brain barrier to deliver therapeutic payloads [30]. The inherent stability and low protein-binding affinity of the hydroxyl surface [31] make them ideal candidates for systemic administration in CNS disorders and demonstrate their successful applications across diverse fields, from drug delivery to catalysis [32]. Our PAMAM hydroxyl dendrimers specifically localize in activated/dysregulated microglia in various animal models, including a mouse model of RTT [22,23,25]. Several dendrimer–drug conjugates are in clinical trials: D-N-acetylcysteine (D-NAC) improved survival and decreased inflammation and injury in phase 2a clinical trials for severe COVID-19; and D-4517.2 conjugate improved outcomes in age-related macular degeneration and diabetic macular edema, diminishing the need for intravitreal aflibercept (EYLEA^R^) injections [33,34]. Our previous research showed that D-JHU29, a glutaminase antagonist conjugated to a dendrimer, represents a viable strategy for selectively inhibiting microglial glutaminase activity. It specifically inhibited microglial glutaminase and improved the neurobehavioral phenotype in *Mecp2* KO mice, demonstrating that inhibiting microglial glutaminase is a potentially promising strategy in RTT [22]. We recently synthesized another dendrimer conjugate, D-TTM020, using a structurally distinct and potent glutamine antagonist by attaching the DON prodrug TTM020 to the dendrimer and compared it with D-JHU in a mouse model of chronic social defeat-induced stress (CSDS) [26,35]. Both D-TTM020 and D-JHU29 alleviated stress and social avoidance in CSDS mice; however, D-TTM020 also alleviated anxiety-like behavior and improved recognition memory, demonstrating its superiority over D-JHU29 [26].

Based on this, we evaluated the efficacy of D-TTM020 in a translationally relevant female *Mecp2* heterozygous (het) mouse model of RTT. We show that D-TTM020 precisely targeted activated/dysregulated glia in *Mecp2* null and *Mecp2* het mice at a low dose and improved the overall neurobehavioral phenotype compared to free TTM020. The co-localization of the dendrimer, conjugated to the Cy5 fluorophore, demonstrates targeting to microglia in pathology-relevant areas in *Mecp2* female het mice. We determined the optimal D-TTM020 dosage based on the extent of microglial glutaminase inhibition in the severe *Mecp2* KO mouse model. Then, we tested the efficacy of the optimized dose in het mice. We show that D-TTM020 significantly improved neurobehavioral scores in symptomatic *Mecp2* het mice, accompanied by decreased inflammation and increased synaptic protein levels, effects not observed with the free drug. Our findings represent a crucial step in advancing therapies for RTT, demonstrating clinical potential as a selective, cell-targeted strategy. We demonstrate precise delivery to microglial cells and robust functional improvements, particularly in the symptomatic, clinically relevant female het model, offering a clear path toward developing an effective and targeted therapeutic strategy for RTT patients.

## 2. Materials and Methods

### 2.1. Animals

We bred female *Mecp2*^tm1.1Bird−/+^ het mice (Jackson Laboratories, Bar Harbor, ME, USA, RRID: IMSR_JAX:003890) with wild-type (WT) male mice for all the experiments unless otherwise stated. All animals were on a C57BL/6 background. Starting at 2.5 months of age, the het phenotype was screened for symptoms (abnormal locomotion, paw clench), and animals were included in the study when the overall neurobehavior score was >2. For dose optimization studies, the *Mecp2* het mice were crossed with CX3CR1^GFP/GFP^ males (Jackson Laboratories, Bar Harbor, ME, USA, RRID: IMSR_JAX:005582), resulting in *Mecp2* KO CX3CR1^GFP/+^ pups. We housed 5 mice per cage in the animal facility under a 14 h on/10 h off light cycle. The animals had free access to food and water. The Johns Hopkins Animal Care and Use Committee (MO21M208, 8 August 2021) approved all protocols and procedures and followed ARRIVE guidelines [36].

### 2.2. Treatment and Assessment Paradigm

#### 2.2.1. Dose Optimization Studies in *Mecp2* KO Male Mice

Glutaminase activity: To assess in vivo efficacy, we first conducted a dose–response study in male KO mice and determined the optimal doses of D-TTM020 for long-term efficacy studies in MeCP2-deficient mice. We utilized the WT and KO mice with het GFP-labeled CX3CR1 (chemokine receptor constitutively found on microglia) (CX3CR1^GFP/+^ WT M, CX3CR1^GFP/+^ KO M, respectively). CX3CR1^GFP/+^ KO mice were treated with D-TTM020 at 0.3 mg/kg and 1 mg/kg (TTM020 basis) given intraperitoneally (IP) at the age of 5–6 weeks, when phenotypic changes begin to manifest. Control CX3CR1^GFP/+^ KO and CX3CR1^GFP/+^ WT mice were treated with saline. Animals were euthanized 24 h after treatment, saline perfused, and their brains were harvested. We then prepared single-cell suspensions for fluorescent-activated flow cytometry (FACS). GFP^+^ microglia were collected via FACS and analyzed for glutaminase activity using a commercially available glutaminase activity assay kit (K455-100; Bio Vision, Waltham, MA, USA).

Composite neurobehavioral scores: The *Mecp2* KOs (3 weeks old) were randomly assigned using the block randomization method to three experimental groups treated weekly with (1) saline (0.9% *w*/*v* NaCl injection, USP, NDC 00409-4888-10, Hospira Inc., a Pfizer company, Lake Forest, IL, USA) or (2) an optimal dose of D-TTM020 (1 mg/kg IP). WT littermates were treated with saline (0.9% *w*/*v* NaCl) to compare behavioral phenotypes. To evaluate therapeutic efficacy, 100 μL of each formulation, prepared in sterile saline, was injected via the IP route twice weekly for 3 weeks. Experimenters not involved with the assessments or analyses performed drug assignments and injections. WT mice (controls) were injected weekly with PBS. A composite neurobehavior scoring system was used to quantify the RTT-like phenotype, assessing respiratory function, gait, general mobility, and bilateral hind-paw clenching. Severity in each domain was graded from 0 to 3, with 0 as normal. For inclusion in this study, *Mecp2* KO mice with a symptomatic baseline score > 2 (paw clenching score > 0) were included in the treatment arms. Treatment efficacy was determined by comparing endpoint scores with baseline levels, with all video-recorded behavior analyzed by a blinded evaluator to minimize observer bias [25].

#### 2.2.2. Biodistribution in Symptomatic *Mecp2* Het Mice

Symptomatic *Mecp2* het females and age-matched healthy WT littermates were injected with PAMAM hydroxyl dendrimer conjugated with Cy5 fluorophore (10 mg/kg on a dendrimer basis, administered IP) [35]. Following anesthesia with isoflurane, mice underwent transcardial perfusion with ice-cold 1X PBS. The brains were harvested and post-fixed overnight. Cryoprotection was performed in a stepwise manner, initially 15% sucrose overnight, followed by 30% sucrose overnight for 16 h at 4 °C, and frozen in dry ice. The tissue was sectioned using a cryostat (Leica CM 1950, Leica Biosystems Cryostats, Nussloch, Germany) at 30 μm and mounted onto gelatin-coated glass slides to examine the distribution of Cy5-conjugated PAMAM hydroxyl dendrimer in various brain areas of *Mecp2* symptomatic hets.

#### 2.2.3. Immunohistochemistry and Confocal Imaging

Immunohistochemistry was performed to examine the brain distribution of Cy5-conjugated PAMAM hydroxyl dendrimer of *Mecp2-symptomatic heterozygous* mice. Tissue sections (30 μm thick) were stained as per our previous protocol with slight modifications. Briefly, sections were rehydrated in 1× TBS for 10 min at room temperature (RT). Tissues were blocked for 1 h and 15 min at RT in a solution containing 0.1% TritonX-100/TBS (80%), normal donkey serum (10%), and 3% BSA/PBS (10%). We incubated the sections at 4 °C in the blocking solution with primary antibody rabbit anti-Iba-1 (1:250, Cat 019-19741, Fujifilm Wako Pure Chemical Corp, Osaka, Japan). Following three washes in 1× TBS for 10 min at room temperature, the tissue was then incubated in a blocking solution containing secondary antibody Alexa Fluor 488 Donkey anti-Rabbit (Jakson ImmunoResearch; Cat 711-545-152, West Grove, PA, USA) at RT for 2 h.

Tissue was rinsed 3× at RT in 1× TBS, in the last wash, tissue was incubated in DAPI (1:1000)/1× TBS for 10 min and rinsed in 1× TBS. The slide was coverslipped with Prolong Gold Antifade mounting media and #1.5 coverslips. Sections were imaged with a Leica SP8 confocal microscope with HyD detectors (Leica Biosystems, Nussloch, Germany). Low-magnification images were acquired with a 20× objective (HC PL APO CS2 20X/0.75 DRY, Nussloch, Germany). For the 100× high magnification, a 63× (HC PL APO CS2 63X/1.40 OIL, Nussloch, Germany) objective was used with a zoom function of 1.59. High-magnification images were collected as a Z-stack (16 steps and 2 μm step size) and are shown as a max projection. Co-localization was confirmed with an orthogonal section of the Z-stack.

#### 2.2.4. Efficacy Studies in Female *Mecp2* Het Mice

Experimental Paradigm: Based on the experiments conducted in KO mice, we determined the optimal dose of D-TTM020. Subsequently, we conducted efficacy studies in symptomatic 16-week-old female *Mecp2* het mice, as these mice are more representative of the patient population in terms of severity and lifespan. Initially, we screened these het mice and confirmed the presence of RTT symptoms through a baseline neurobehavioral scoring. We scored using a composite neurobehavioral score (NBS) that encompasses assessments of mobility, gait, paw clenching, tremors, and respiration, each scored 0–3. Animals with scores exceeding 2 for paw clench, mobility, or gait were considered symptomatic. Similarly to the KO studies, the symptomatic het mice were randomized into three experimental groups using block randomization. Each group received biweekly treatment with saline (0.9% *w*/*v* NaCl), free TTM020 (1 mg/kg), or D-TTM020 (1 mg/kg on a TTM020 basis), dissolved in saline. We administered the treatments intraperitoneally (IP) at a volume of 100 μL and prepared all formulations in sterile saline. WT was injected with an equivalent volume of saline as a control. We conducted behavioral testing at the end of the 8th week, including neurobehavioral scores, open-field testing, and contextual fear conditioning, in littermate cohorts. Contextual fear conditioning was the final assessment due to the higher level of stress involved.

Open field: In the 8th week, mouse activity was assessed in an open-field enclosure (38 × 26.5 cm) for 10 min. *ANY-maze* software (version 7.3, Stoelting Co., Wood Dale, IL, USA) quantified distance traveled and mean speed in the enclosure [25].

Contextual fear conditioning: On day 1, the mice were introduced to a fear-conditioning arena (10.75″ × 9.97″ × 11.25″; Med Associates, St. Albans, VT, USA) and allowed to habituate for two minutes. Animal were habituated for 2 min and then exposed to three tone–shock pairings (2 min apart) for 30 s each, with a 2 s shock (0.7 mV) co-terminating with the tone. On the subsequent day, contextual fear memory was assessed by returning the mice to the original chamber (Context A) for five minutes. One hour later, cued fear memory was evaluated in a modified environment (Context B) where they experienced 2 min of habituation, followed by three tone-only presentations at 1 min intervals. On day three, fear extinction was monitored during a 40 min session involving tone-only presentations. All behavioral data were acquired via videorecording and quantified using *ANY-maze* software (version 7.3, Stoelting Co., Wood Dale, IL, USA) by blinded personnel [25].

#### 2.2.5. Biochemical Analysis

Following 24 h after the last dose, mice were anesthetized and perfused transcardially with 60 mL of PBS (PH = 7.4). Brains were harvested, quickly micro-dissected (cortex, striatum, and hippocampus), and flash-frozen in liquid nitrogen. Equalized protein samples were used on Meso Scale Diagnostics kit (V-PLEX Proinflammatory Panel 1 Mouse Kit, K15048D, MSD, Rockville, MD, USA) or pre-coated ELISA plates and incubated per the protocols. BDNF (Biosensis Mature BDNF Rapid TM enzyme-linked immunosorbent assay kit, BEK-2211, Thebartan, SA, Australia) and PSD-95 (mouse PSD-95 assay kit, A303515,antibodies.com, Cambridge, UK) levels were measured as per kit instructions.

### 2.3. Statistical Analysis

Experimental groups were assigned using a randomized block design, and all investigators remained blinded to treatment conditions throughout the study. Sample sizes were determined via a power analysis for GEE nested designs using PASS-14 (power = 0.8, δ = 1), based on preliminary neurobehavioral scoring data. For biochemical assays and dose–response evaluations, groups consisted of n = 3–5 animals, while behavioral assessments had n = 8–15 mice per group. Statistical analyses and data visualization were performed using GraphPad Prism 7.0. Quantitative results are expressed as mean ± SD. For parametric data, one-way or two-way ANOVA followed by Dunnett’s post hoc test was employed. The non-parametric Kruskal–Wallis test was used for neurobehavioral score comparisons. Post hoc analyses were conducted only when the initial F-statistic achieved significance (*p* < 0.05)

## 3. Results

### 3.1. Systemic D-TTM020 Therapy Decreased Microglial Glutaminase Activity and Improved Composite Neurobehavior in Mecp2 KO Mice

**Figure 1:**
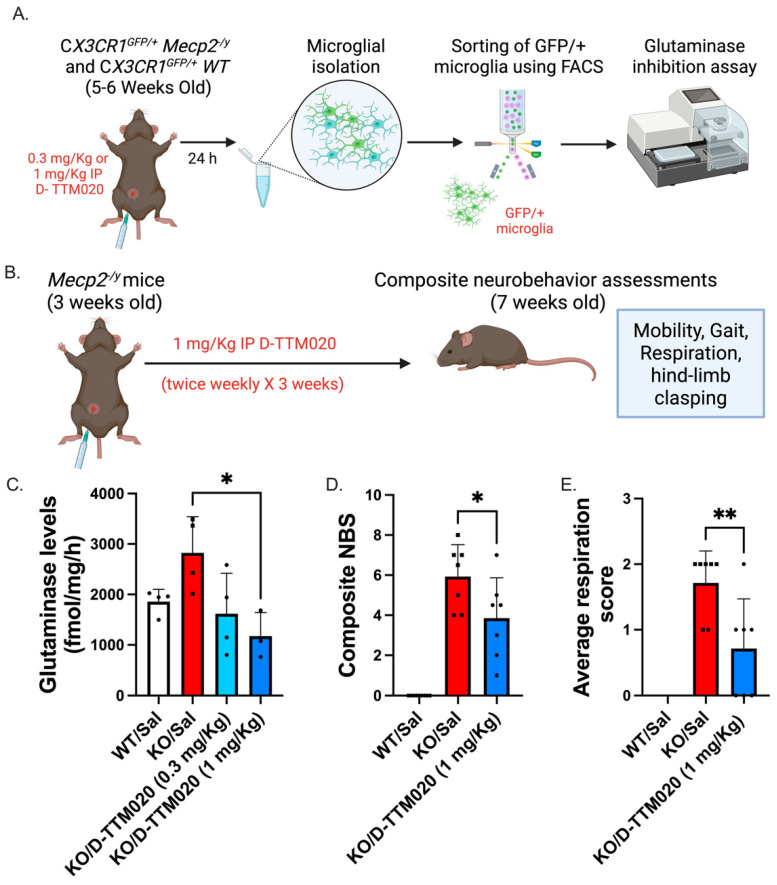
D-TTM020 increases microglial glutaminase activity and improves the neurobehavioral score. (**A**) Experimental paradigm for the dose optimization of D-TTM020 and (**B**) neurobehavioral studies using the optimized dose, (**C**) 1 mg/kg IP single dose of D-TTM020 reduces microglial glutaminase levels 24 h post-treatment in CX3CR1*^GFP/+^ Mecp2^y^* male KO mice. D-TTM020 (1 mg/kg IP) improves (**D**) composite neurobehavioral phenotype and (**E**) respiration in *Mecp2*^/*y*^ male KO mice. * *p* < 0.05, ** *p* < 0.01 versus KO/Sal. Data is represented as mean ± SD, n = 3–7/group.

We first determined the optimal dose to inhibit microglial glutaminase activity using symptomatic CX3CR1^GFP/+^ *Mecp2* KO and CX3CR1^GFP/+^ WT mice (Figure 1A,B). The *Mecp2* KO mice (5–6 weeks old) with a baseline score > 2, including a paw-clench and immobility score > 0, were included in the study. We treated CX3CR1^GFP/+^ *Mecp2* KO with D-TTM020 (0.3 mg/kg or 1 mg/kg IP; TTM020 equivalent) to determine the optimal dose. After 24 h, the animals were euthanized, and the brains were harvested. GFP^+^ microglia were sorted via FACS, and glutaminase activity was measured using a commercially available glutaminase activity assay kit (Figure 1A). We observed a reduction in microglial glutaminase activity in *Mecp2*-deficient mice with both 0.3 mg/kg and 1 mg/kg IP doses; however, the decrease was significant only at 1 mg/kg. Both 0.3 mg/kg IP and 1 mg/kg IP doses resulted in lower mean glutaminase activity compared to saline-treated KO mice; only the 1 mg/kg dose reached statistical significance (*p* = 0.0273). No significant difference was observed between the 0.3 mg/kg and 1 mg/kg (*p* = 0.09318) groups, suggesting a possible ceiling effect for glutaminase inhibition within this dose range. While the 0.3 mg/kg dose showed a numerical trend toward reduction, it did not consistently meet the threshold for significance across our replicates (*p* = 0.0997).

Based on this, we selected 1 mg/kg IP as an optimal dose for our further experiments (Figure 1C). We next evaluated this dose for short-term efficacy in the *Mecp2* KO mice. We injected 3-week-old KO with D-TTM020 twice weekly for 3 weeks and compared the composite neurobehavior score with saline-treated *Mecp2* KOs and WT littermates at 7 weeks of age (Figure 1B). We observed an initial decrease in body weight during the first week of treatment across both saline and treated groups, which subsequently stabilized, and the treated mice maintained a consistent weight plateau for the remainder of the study period. Mean WT weight was recorded as (23.28 ± 1.647 g) at the end of the study. No significant differences in body weight were observed between het saline (mean ± SD; 15.93 ± 1.59 g), TTM020 (1.463 ± 0.629 g) and D-TTM020 (15.23 ± 1.858 g) treated mice at the study endpoint. However, we observed a significant decrease in neurobehavioral scores, particularly in respiration scores, indicating an improvement in the RTT phenotype with the D-TTM020 therapy (Figure 1D,E). Based on this, the D-TTM020 dose of 1 mg/kg IP was used to evaluate efficacy in clinically relevant female *Mecp2* het mice.

### 3.2. PAMAM Hydroxyl Dendrimers Precisely Targeted ‘Dysregulated’ Microglia in Symptomatic Mecp2 Het Mice

**Figure 2:**
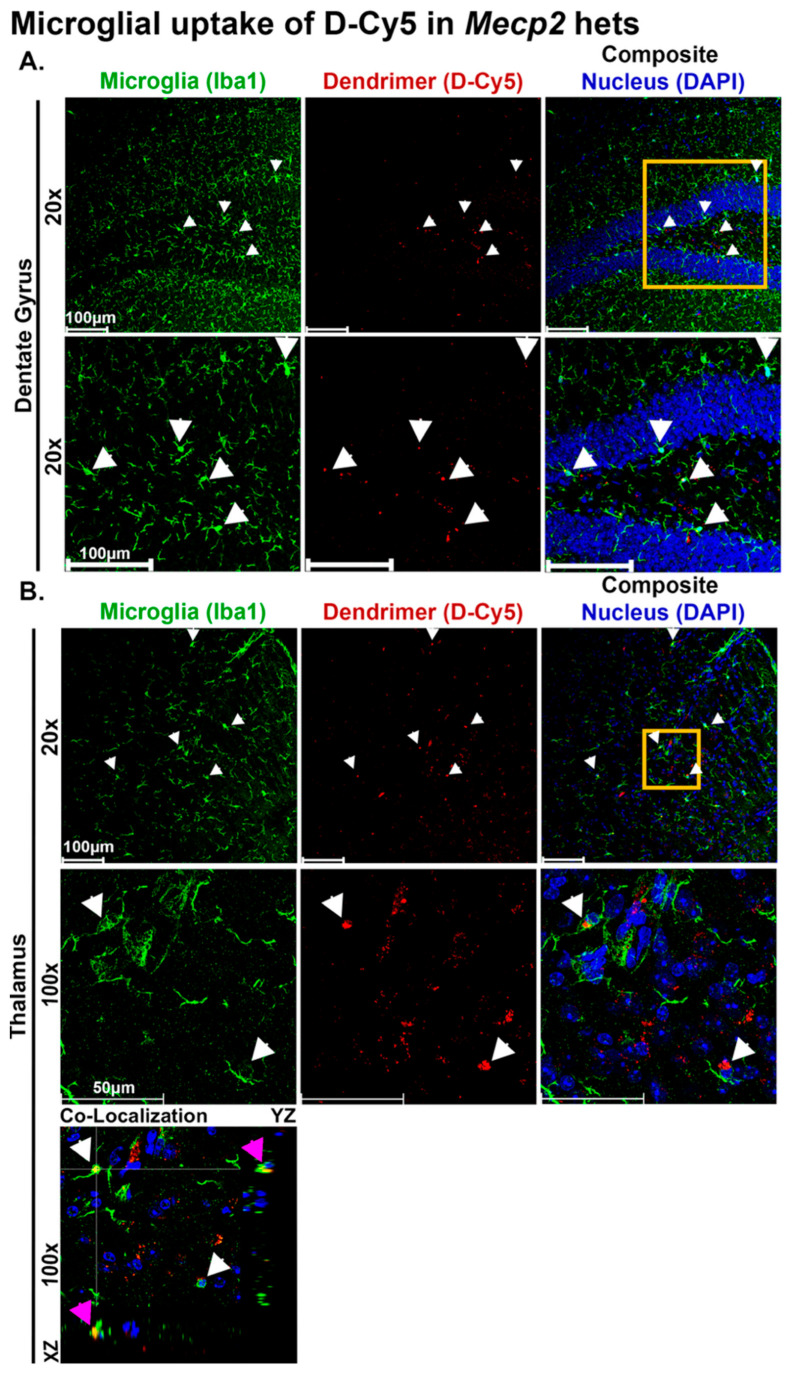
Microglial uptake of D-Cy5 in the thalamus and dentate gyrus region of hippocampus. Symptomatic *Mecp2* hets were administered D-Cy5 (10 mg/kg, IP), and brains harvested 24 h later to evaluate cellular co-localization. Cy5-conjugated hydroxyl dendrimer (shown as red) colocalizes with microglia (Iba1, shown as green) in the (**A**) dentate gyrus region of hippocampus (20×), white arrows show microglial uptake of D-Cy5 and the yellow square denotes the area zoomed-in the second row and (**B**) thalamus (20× and 100×). White arrows show microglial uptake D-Cy5, the yellow square represents the area imaged at higher resolution (100×) in the second row. Image shown is a maximum-projection of a Z-stack (16 steps and 2 μm step size). Third rows show orthogonal section of the Z-stack from the thalamus region to show co-localization of the dendrimer in microglia (white arrows show the cells that have up taken the dendrimer). XZ (bottom) and YZ (right) planes of the orthogonal section show the co-localization of the dendrimer in the microglia, the magenta arrows show the microglia selected for the orthogonal section.

We previously showed that microglia in symptomatic het mice exhibit immune dysregulation, characterized by increased proinflammatory cytokine expression [23]. Here, we show that fluorescent conjugated hydroxyl dendrimer (D-Cy5) administered 10 mg/kg IP crosses the blood–brain barrier and colocalizes in Iba-1 positive microglia in the dentate gyrus and thalamus (Figure 2), which are brain regions known to be associated with Rett pathology in this model.

### 3.3. D-TTM020 Improved the Neurobehavioral Phenotype in Mecp2 Het Mice

#### 3.3.1. Composite Neurobehavior

For these experiments, 16-week-old WT- and het mice were used, since we previously demonstrated that het mice at this age are symptomatic and exhibit pathophysiological changes, making this a clinically relevant time point, as seen in girls with RTT [37]. Baseline scores for mobility, gait, and paw clenching were obtained on a scale of 0–3, as described in methods for the composite NBS. Mice with scores greater than 2 for paw clenching, mobility, or gait were considered symptomatic.

**Figure 3:**
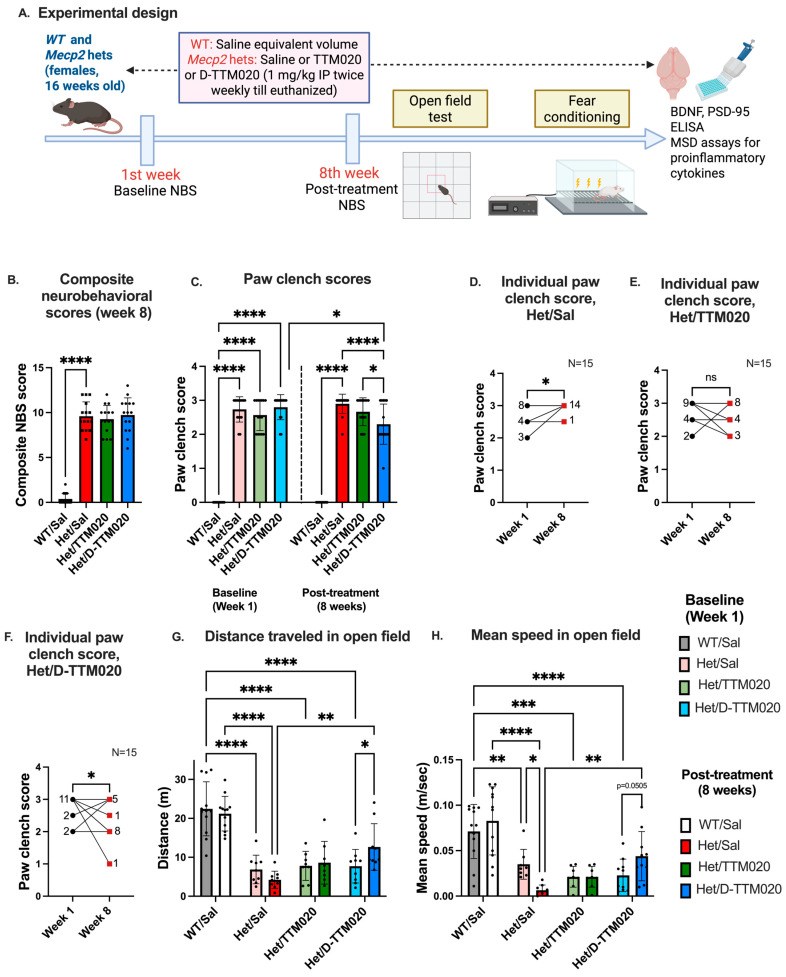
D-TTM020 improves neurobehavioral deficits in *Mecp2* hets (**A**) Experimental paradigm, (**B**) Composite neurobehavioral scores after 8 weeks of treatment, and (**C**) change in paw-clench score; baseline versus the 8th week of treatment, D-TTM020, but not free TTM020, reduced the paw clench in the *Mecp2* hets over time, (**D**–**F**) individual paw-clench scores at 1st week versus the 8th week in different groups. (**D**) Hets significantly deteriorated over time. (**E**) TTM020 did not improve the paw-clench score after 8 weeks of treatment, but (**F**) D-TTM020 significantly improved the scores at the 8th week (*p* < 0.05). Each graph displays the individual scores corresponding to the number of animals before and after treatment, (**G**) systemic administration of D-TTM020 over 8 weeks improved overall activity in the open-field compared with their pretreatment baseline (week 1), and versus the saline group at 8th week. (**H**) The improvement in mean speed validates increased activity and mobility in the open field. Data is represented as mean± SD. **** *p* < 0.0001, *** *p* < 0.001, ** *p* < 0.01, * *p* < 0.05, ns: not significant. n = 15/group for NBS and n = 8–9/group for open-field activity.

To maintain clinical and translational relevance, only symptomatic het mice were randomized into the treatment groups and treated twice weekly for 8 weeks with saline or 1 mg/kg IP of TTM020 or D-TTM020. Age-matched WT mice received saline twice weekly. Neurobehavioral phenotypes and behavior were examined at the end of the eighth week. Although there was no change in the overall neurobehavioral score over this time, a significant improvement in the paw-clench score, which directly correlates with the motor phenotype, was observed in *Mecp2* het mice treated with D-TTM020, but not with saline- or free-drug treatment (Figure 3B,C).

We further analyzed the distribution of treatment responses in Figure 3D–F and performed a responder analysis for the phenotypic scores. Improvement was defined as a score reaching a threshold of at least 1 standard deviation (SD) better than the baseline mean. The threshold was calculated as the baseline mean-1X standard deviation. All hets showed poor phenotype at the eighth week. In the TTM020 group, 20% of the mice demonstrated improvement, compared to 80% animals that showed deterioration in the eighth week. Conversely, while 100% of the saline-treated mice showed same or worsened symptoms over the study period, this was reduced to 33.3% in the D-TTM020-treated group, where >60% of the animals showed improvement in paw-clench scores relative to their pretreatment baseline. These data indicate that D-TTM020 provided a more consistent improvement in paw clenching compared to the free drug. Each graph displays the individual scores corresponding to the number of animals (Figure 3D–F).

#### 3.3.2. Open-Field Test

We assessed motor function by recording the mice in an open field; each mouse was placed in a clean, standard housing cage, and activity was recorded for 10 min. Het mice treated with D-TTM020 exhibited an increase in the total distance traveled in comparison to their pretreatment baseline (week 1), unlike the TTM020 group. D-TTM020 treatment also showed significant difference with the saline-treated het group at the eighth week, demonstrating improved locomotor activity and exploratory behavior (Figure 3G).

At the end of the eight-week treatment, we also observed an increase in mean speed with the D-TTM020 treated group versus their own pretreatment baseline (*p* = 0.0505). This was significantly higher than the saline-treated hets at the eighth week (Figure 3H).

### 3.4. Systemic D-TTM020 Monotherapy Improved Hippocampus-Based Fear Memory

**Figure 4:**
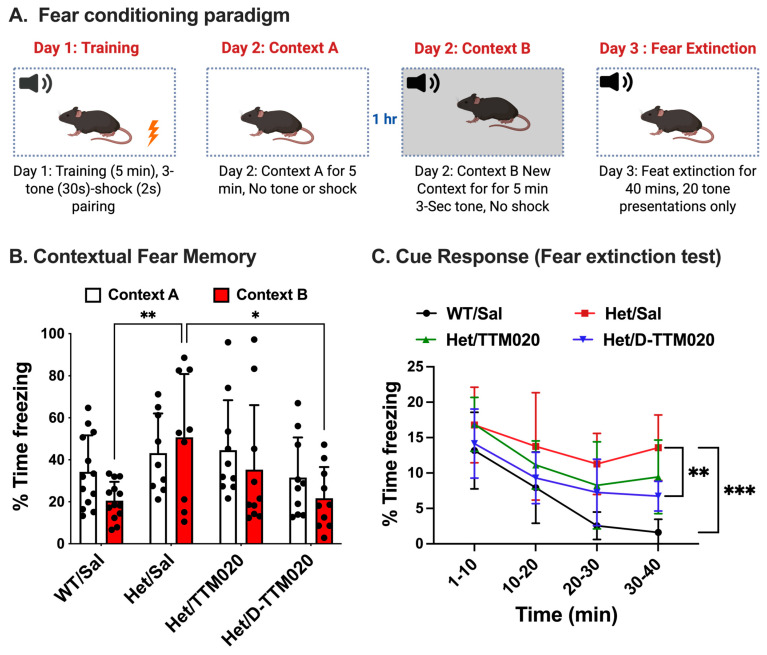
D-TTM020 restores contextual fear memory in *Mecp2* het mice. (**A**) Fear-conditioning paradigm, (**B**) contextual fear memory. D-TTM020-treated hets rescued conditioned fear memory to baseline levels. (**C**) Fear extinction test (Cue response). D-TTM020 improved contextual fear memory and cue response in *Mecp2* hets versus saline and free TTM020. *** *p* < 0.001, ** *p* < 0.01, * *p* < 0.05 in comparison to the saline-treated hets. Data is represented as mean ± SD. n = 9–13/group.

We evaluated WT- and het mice in the contextual fear-conditioning paradigm to assess fear memory, which has previously been demonstrated to be compromised in this mouse model of RTT (Figure 4A) [15,21]. In normal WT mice, freezing behavior is diminished in a novel context (Context B) compared to the training context in which the shock was administered (Figure 4B). Het mice, however, did not exhibit this reduction in fear behavior in the novel context (Context B), indicating an impairment in the fear memory (WT versus het, *p* = 0.0085), specifically impaired context-discrimination.

D-TTM020 (1 mg/kg) administration recovered this behavior, decreasing freezing time in Context B (*p* = 0.0215), as in normal WT mice, but free TTM020 (1 mg/kg IP) did not (Figure 4B). While WT mice naturally decrease freezing behavior in a novel context (Context B), untreated het mice fail to do so, indicating memory impairment. Treatment with D-TTM020 restored this discrimination, free TTM020 also showed some improvement but it did not reach significance at the same dose (*p* = 0.5207); it failed to elicit a significant improvement compared with het mice. We also observed a significant extinction of a cue response with D-TTM020 (*p* = 0.005), but not TTM020 (*p* = 0.265), which had between a 30 and 40 min bin for the fear extinction test, similar to the extinction learning pattern seen in WT mice (Figure 4C). This improvement in extinction learning is not associated with forgetting the original fear response to the shock, but rather with the creation of a new, competing memory that inhibits the expression of the fear response [38]. Our data demonstrate that pathology-based, selective glial targeting of D-TTM020 to specific brain regions is crucial for improving fear memory in RTT.

### 3.5. D-TTM020 Improved the BDNF Levels and Synaptic Plasticity in Mecp2 Het Mice

**Figure 5:**
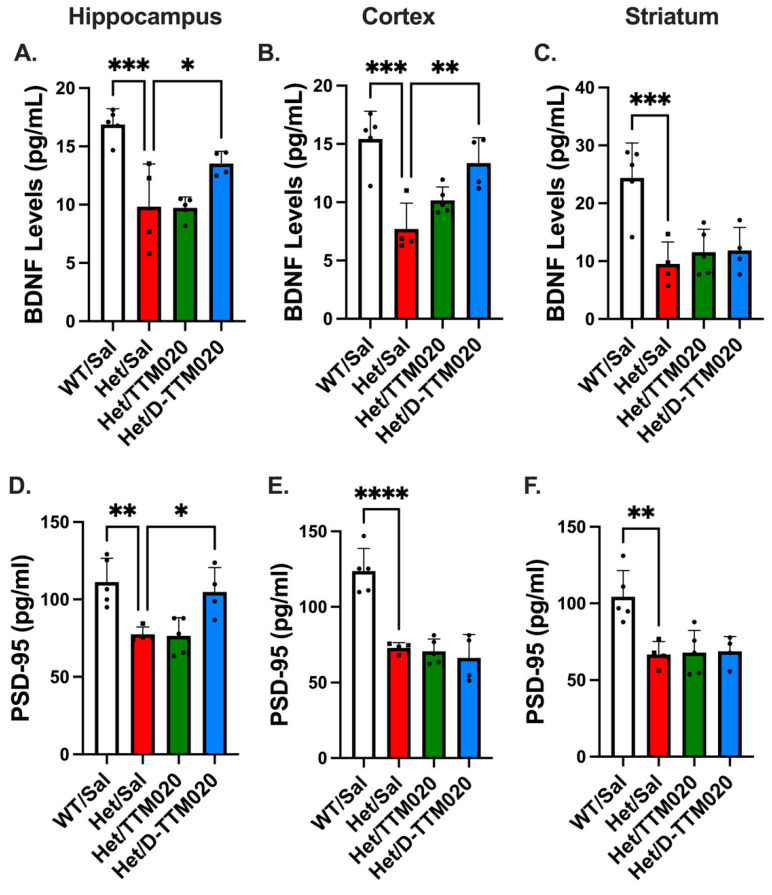
D-TTM020 improved the BDNF levels and synaptic plasticity in chronically treated hets. (**A**–**C**) D-TTM020 treatment increased BDNF levels in the hippocampus and cortex, and (**D**–**F**) PSD-95 levels in the hippocampus, compared with saline-treated and TTM020-treated female het mice. D-TTM020 showed no improvement in PSD-95 levels in the cortex and striatum. Data is represented as mean ± SD. **** *p* < 0.0001, *** *p* < 0.001, ** *p* < 0.01, * *p* < 0.05, n = 3–6/group.

Since we observed D-Cy5 localization in hippocampal areas and an improvement in hippocampal-dependent memory, we next tested whether D-TTM020 also improves BDNF levels and synaptic plasticity in the brain, in a region-specific manner. We tested these in hippocampal, cortical, and striatal brain regions, as they are most closely associated with RTT pathology. After 8 weeks of systemic D-TTM020 treatment, we observed an increase in BDNF protein levels, a crucial synaptic plasticity and memory regulator, in both the cortex and hippocampus of symptomatic het mice. We also observed the improvement in PSD-95 levels in the hippocampus with D-TTM020 therapy. These effects are not observed with TTM020 alone at this dose, suggesting that microglial targeting with D-TTM020 is more effective at increasing BDNF levels and activating PSD-95 expression in the hippocampus of RTT mice, which also explains the improvement in learning in the fear extinction test (Figure 5).

### 3.6. D-TTM020 Attenuated Proinflammatory Cytokine Levels in the Hippocampus and Cortex of Mecp2 Het Mice

**Figure 6:**
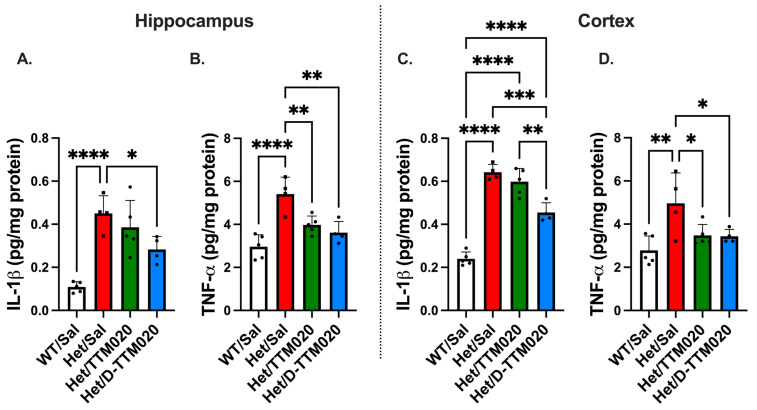
D-TTM020 reduced proinflammatory cytokines in the hippocampus and cortex of female het mice. (**A**,**B**) TTM020 reduced IL-β and TNF-α levels in the hippocampus and (**C**,**D**) the cortex of *Mecp2* het mice. Data is represented as mean ± SD. **** *p* < 0.0001, *** *p* < 0.001, ** *p* < 0.01, * *p* < 0.05, n = 3–6/group.

The activation of glia and the resulting neuroinflammation are involved in the pathogenesis and symptomatic worsening of *Mecp2*-deficient models [11,25]. We have previously shown that microglia exhibit immune dysregulation in this model and contribute neuronal inflammation in the symptomatic het mice [23,25]. After 8 weeks, D-TTM020 significantly lowered IL-1β levels in the cortex (*p* = 0.0001) and hippocampus (*p* = 0.0317). However, TTM020 showed no significant decrease in either region (*p* = 0.5211 and *p* = 0.3522 for the hippocampus and cortex, respectively). The difference between the two treatments in the cortex was highly significant (*p* = 0.0018). Both D-TTM020 and free TTM020 successfully mitigated levels of the proinflammatory cytokine TNF-α in the hippocampus and cortex, but D-TTM020 demonstrated a significantly superior reduction (*p* = 0.0015 and *p* = 0.0384 for hippocampus and cortex) (Figure 6). These results strongly support D-TTM020 as a significantly superior therapeutic strategy for targeting neuroinflammation in the *Mecp2* het mice.

## 4. Discussion

Disruption of glutamate neurotransmission, a hallmark of RTT, alters glutamate homeostasis, contributing to synaptic dysfunction [13,39,40]. Systemic glutamine antagonists possess the potential to mitigate excitotoxicity [41], yet their clinical application in the management of CNS disorders is hindered by their limited ability to traverse the BBB effectively. This poor penetration necessitates high- and frequent doses, leading to non-specific inhibition and undesirable side effects [18,19,20].

The drug DON, a potent glutamine antagonist, initially showed promise in clinical trials but was abandoned because it demonstrated dose-limited adverse effects [17]. We previously conjugated a DON prodrug, TTM020, to a PAMAM hydroxyl dendrimer for targeted microglial glutamine delivery and evaluated its therapeutic potential in a mouse model of CSDS [26,35]. D-TTM020 demonstrated enhanced CNS targeting and mitigated the severe gastrointestinal-related toxicities associated with free TTM020 in the CSDS model [26]. Similarly to CSDS pathology, RTT pathogenesis also involves significant microglial activation, neuroinflammation, and excessive glutamatergic signaling [15,22,42]. Therefore, we determined whether D-TTM020 can effectively attenuate microglia-driven excitotoxicity and neuroinflammation, thereby rescuing the associated behavioral- and memory deficits in the RTT model. Here, we present evidence that D-TTM020 specifically targets microglial glutaminase, enhances overall neurobehavioral and synaptic plasticity, and simultaneously reduces neuroinflammation in a clinically relevant model of RTT. Our dendrimer nanotherapeutic approach addresses critical limitations of highly potent glutamine antagonists, such as poor blood–brain barrier penetration, enabling precise targeting of activated microglia at lower doses. Our lab has previously shown dendrimer localization in activated microglia and astrocytes across various disease models [24,35,37]. We now report a microglial uptake of D-Cy5 in the thalamus and DG regions of the hippocampus, which is also associated with improved fear memory and synaptic plasticity in D-TTM020-treated symptomatic *Mecp2* het mice.

*Mecp2* KO microglia secrete significantly higher levels of glutamate compared to WT mice, primarily due to the increased expression of glutaminase in vitro [42]. We specifically examined microglial glutaminase levels in KO mice treated with two doses of D-TTM020 (0.3 and 1 mg/kg on a TTM020 basis) in comparison to saline-treated KO and WT littermates. These KO male mice lack the MeCP2 protein entirely, resulting in a severe phenotype and rapid disease-progression, which makes them an ideal model for identifying a therapeutic window and dose-responsiveness [43,44,45,46]. By using the more severe model for dose-finding, we ensured that any potential for dose-dependent rescue could be detected clearly and efficiently at this stage. The 0.3 mg/kg IP dose reduced microglial glutaminase levels, but we observed a greater reduction with the 1 mg/kg IP dose. It exhibited a superior dose response, prompting the selection of a 1 mg/kg dose for further experiments. Over three weeks, the twice-weekly administration of D-TTM020 improved total neurobehavioral scores and respiratory function, as evidenced by more regular breathing patterns in symptomatic KO mice. It was assessed as part of the composite neurobehavioral score to capture gross phenotypic manifestations of *Mecp2* deficiency. While this qualitative scoring does not substitute for the physiological precision of whole-body plethysmography, it is a robust indicator of clinically overt respiratory dysfunction and overall morbidity in the mice. These irregular breathing patterns are prevalent in RTT patients and can be fatal or lead to the need for advanced respiratory support in these patients [47]. Once an optimal dose was established in the KO model, we transitioned to the translationally relevant female hets for the efficacy study, which more closely recapitulate the genetic and phenotypic landscape of RTT patients. This allowed us to test the efficacy of our optimized dose in a clinically relevant set-up.

We confirmed the uptake of Cy5-conjugated dendrimer (D-Cy5) in symptomatic het mice. Cy5-conjugated dendrimer colocalized with microglia in the striatum, cortex, hypothalamus, thalamus, and red nucleus, but not in healthy littermates. Dysfunction of the striatum, along with the thalamus and hippocampus, plays a significant role in RTT pathogenesis [40,48]. Imaging data showed the localization of the dendrimer with microglial cells in these areas, showcasing the therapeutic potential of dendrimer therapy for RTT at this dose. We then conducted efficacy studies using 1 mg/kg of D-TTM020 therapy in het mice. We observed an overall improvement in composite neurobehavior, particularly paw clenching and mobility, as reflected in improved locomotor activity and speed in the open field with D-TTM020 but not with the free drug. This indicates that D-TTM020 therapy at 1 mg/kg on a TTM020 basis specifically targeted microglial glutaminase and consistently improved neurobehavioral phenotypes in these mice, which the free TTM020 could not. Interestingly, while D-TTM020 treatment significantly improved respiratory scores in KO male mice, a similar effect was not observed in the female hets group. This sex-specific difference likely reflects the mosaic expression of MeCP2 in females due to X-chromosome inactivation, resulting in a milder, more variable respiratory phenotype compared to the severe, uniform deficits seen in male null mice. Consequently, the female hets may require longer treatment durations or more sensitive physiological measurements, such as plethysmography, to resolve the minute changes in breathing patterns. These efficacy results show a strong translational link to previously established findings in the CSDS model and substantiate that targeting microglial glutamatergic pathways is a therapeutic strategy for diverse CNS disorders characterized by neuroinflammation [26,35].

MeCP2 deficiency leads to learning and memory deficits, especially fear memory, due to abnormal neural circuitry in the hippocampus and amygdala [49,50]. The *Mecp2-*deficient hippocampus releases excessive glutamate, impairing spatial cognition and fear extinction [51]. Given the dense connections between the hippocampus and amygdala, excessive glutamate may lead to increased amygdala activation that can alter stress and fear responses and impair memory [25,50]. Cue-dependent memory retrieval was observed in D-TTM020-treated het mice versus saline-treated hets. On day 3, a fear-extinction learning response was observed with D-TTM020, suggesting reduced fear after repeated presentations of a conditioned stimulus (tones) without an unconditioned aversive stimulus (shocks). The hippocampus is involved in formation of episodic memories [52,53] and is necessary for both contextual and cue-based fear conditioning [54]. Based on this, we examined synaptic plasticity and its mechanisms in the hippocampus. PSD-95 regulates synaptogenesis, and therefore, also memory [55,56]. In vivo, D-TTM020 treatment for 8 weeks increased PSD-95 and BDNF levels in the hippocampus of Rett mice, but free TTM020 did not enhance synaptic plasticity in chronically treated het mice. D-TTM020 improved BDNF but not PSD-95 levels in the cortex of these mice and established a clear relationship between molecular recovery and behavioral improvement.

In line with previous studies associating BDNF and PSD-95 with contextual fear extinction, our data showed that D-TTM020 affects the extinction response, likely via BDNF signaling, activating PSD-95 expression in RTT [57,58,59]. This alleviates RTT symptoms, reverses behavioral impairments, and improves hippocampal-dependent memory, as observed in fear-conditioning tests [55,60].

MeCP2 deficiency leads to abnormal glial immune responses, driving neuroinflammation that worsens RTT symptoms [61,62]. MeCP2-deficient microglial cells produce proinflammatory cytokines, glutaminase, and the glutamine transporter SNAT1, causing ongoing injury [10,11,23,63]. Increased proinflammatory cytokine levels have been found in *Mecp2* null- and het mice. Treatment with D-TTM020 reduced proinflammatory cytokines in brain areas controlling behavioral alterations in adult female *Mecp2* het mice. D-TTM020, but not TTM020, therapy improved the neuroinflammatory burden and phenotypic features in mouse models, suggesting that dendrimer conjugation is effective for CNS drug delivery and targeting glia. Both TTM020 and D-TTM020 reduced TNF-α levels in the hippocampus and cortex; however, D-TTM0202 showed a significant reduction in hippocampal and cortical IL-1β levels. This suggests that free TTM020 possesses intrinsic potency to reduce broad markers, such as TNF-α; however, it does not significantly reduce IL-1β, a master regulator of neuroinflammation and a known disruptor of long-term potentiation [64,65]. By sequestering in activated glia and suppressing the IL-1β pathway more effectively than the parent drug, D-TTM020 provides a more targeted and potent neuroprotective effect, translating into behavioral rescues (highly sensitive to IL-1β-mediated synaptic interference) observed in this study.

We have previously demonstrated that mixed glial cultures from *Mecp2* null mice exhibit upregulation and release of proinflammatory cytokines [23]. The brain region-specific microglial alterations and changes in synaptic connectivity led to neuronal pathology in RTT [66,67]. Chronic D-TTM020 treatment enhanced the phenotype, reduced proinflammatory cytokine production, and strengthened synaptic circuitry in the hippocampus, thereby explaining the improvement in fear-extinction memory. The animals showing the most significant functional gains, specifically, less paw clenching and improved contextual fear memory, were the same animals that exhibited the lowest levels of proinflammatory cytokines in the hippocampus. It shows that the anti-inflammatory response of D-TTM020 was the initiating step that allowed the restoration of BDNF, which in turn directly correlated with the stabilization of synaptic structure via PSD-95 and improved hippocampal-dependent memory. We also observed the accumulation of D-Cy5 in the DG region of the hippocampus, a brain region associated with hippocampal-dependent extinction of fear memory [68].

In conclusion, our data suggest that dendrimer-based nanotherapies offer a promising targeted approach to address glutaminergic dysfunction in RTT. While further studies are required to bridge the translational gap between RTT mouse models and patients, these findings suggest that dendrimer-based nanotherapies may stabilize or alter the clinical trajectory of the disease.

## Data Availability

The original contributions presented in this study are included in the article. Further inquiries can be directed to the corresponding author.
